# Role of endothelial colony forming cells (ECFCs) Tetrahydrobiopterin (BH4) in determining ECFCs functionality in coronary artery disease (CAD) patients

**DOI:** 10.1038/s41598-022-06758-8

**Published:** 2022-02-23

**Authors:** Atanu Sen, Archna Singh, Ambuj Roy, Sujata Mohanty, Nitish Naik, Mani Kalaivani, Lakshmy Ramakrishnan

**Affiliations:** 1 Department of Cardiac Biochemistry, All India Institute of Medical Sciences, New Delhi, India; 2 Department of Biochemistry, All India Institute of Medical Sciences, New Delhi, India; 3 Centre of Excellence for Stem Cell Research, All India Institute of Medical Sciences, New Delhi, India; 4 Department of Cardiology, All India Institute of Medical Sciences, New Delhi, India; 5 Department of Biostatistics, All India Institute of Medical Sciences, New Delhi, India

**Keywords:** Biochemistry, Cell biology, Stem cells, Cardiology, Pathogenesis

## Abstract

Nitric oxide (NO^**.**^) is critical for functionality of endothelial colony forming cells (ECFCs). Dimerization of endothelial nitric oxide synthase (eNOS) is must to produce NO^**.**^ and tetrahydrobiopterin (BH4) plays a crucial role in stabilizing this state. We investigated BH4 level in ECFCs and its effect on ECFCs functionality in CAD patients. Intracellular biopterin levels and ECFCs functionality in terms of cell viability, adhesion, proliferation, in vitro wound healing and angiogenesis were assessed. Guanosine Triphosphate Cyclohydrolase-1 (GTPCH-1) expression was studied in ECFCs. Serum total reactive oxygen/nitrogen species was measured and effect of nitrosative stress on ECFC’s biopterins level and functionality were evaluated by treating with 3-morpholino sydnonimine (SIN-1). BH4 level was significantly lower in ECFCs from CAD patients. Cell proliferation, wound closure reflecting cellular migration as well as in vitro angiogenesis were impaired in ECFCs from CAD patients. Wound healing capacity and angiogenesis were positively correlated with ECFC’s BH4. A negative effect of nitrosative stress on biopterins level and cell functionality was observed in SIN-1 treated ECFCs. ECFCs from CAD exhibited impaired functionality and lower BH4 level. Association of BH4 with wound healing capacity and angiogenesis suggest its role in maintaining ECFC’s functionality. Oxidative stress may be a determinant of intracellular biopterin levels.

## Introduction

Coronary artery disease (CAD) is the most prevalent type of cardiovascular diseases (CVDs) and leading cause of death worldwide. Damage of vascular endothelium is the first step which initiates a cascade leading to the development of CAD^1^. The repair and maintenance of vascular endothelium is critical and is not only dependent on proliferation of local endothelial cells (ECs) but also on bone marrow derived endothelial progenitor cells (EPCs)^2^. EPCs have been shown to inversely associate with coronary artery disease severity and is a strong predictor of multivessel CAD after age^3^. Circulating EPC levels also predicts severe endothelial dysfunction in CAD patients independent of classical cardiovascular risk factors^4^. Endothelial nitric oxide synthase (eNOS) derived NO^**.**^ plays a critical role in mobilization EPCs from bone marrow and their recruitment at the site of vascular injury^5^. In vascular disease state, oxidative stress increases and eNOS derived endothelial nitric oxide (NO^**.**^) bioactivity diminishes resulting in endothelial dysfunction. Tetrahydrobiopterin (BH4) the cofactor of eNOS plays a crucial role in maintaining the enzymatic “coupling” state of eNOS as well as biosynthesis of NO^**.**^. The balance between NO^**.**^ and superoxide production by eNOS seems to be dependent on bioavailability of BH4, whereas dihydrobiopterin (BH2), the oxidized form of BH4 does not act as cofactor of eNOS, rather competes with BH4 for eNOS binding which further leads to enzyme uncoupling and superoxide production^6^.

In vitro and in vivo studies on diabetic subjects have shown that depletion of BH4 levels either by decreased expression of guanosine triphosphate cyclohydrolase-1 (GTPCH-1), the rate limiting enzyme of BH4 biosynthesis, or oxidative loss of BH4 leads to increased production of superoxide and peroxynitrite, which in turn affect EPC number as well as cellular functionality^7^. Recent clinical study in patients with primary hyperaldosteronemia (PHA), in patients with hypertension and in over weight postmenopausal subjects indicate the depletion of intracellular BH4 levels by oxidative degradation and or downregulation of GTPCH1/BH4 pathway with an associated reduction in number and functionality of EPCs^8^. Direct evidence from DOCA-salt induced hypertensive mice suggest that oxidative loss of BH4 is enough to induce eNOS uncoupling^9^. Similarly, in vivo study in diabetic mice confirmed that, increased endothelial BH4 by transgenic GTPCH overexpression was partially able to correct eNOS uncoupling, but the ratio of BH4 to BH2 was largely unaffected^10^. However, most studies on understanding the role of BH4 in maintaining catalytically active state of eNOS and thereby generation of NO^**.**^ in vascular disease states like diabetes and hypertension, has focused on early EPCs. Recent studies have suggested that early EPCs are not directly involved but only support angiogenesis through paracrine signals whereas that late EPCs also known as endothelial colony forming cells (ECFCs) are the only known endothelial precursor and are directly involved in the process of neovascularization or reendothelization^11^ and therefore may be more relevant to study in the context of vascular diseases. The effect of intracellular BH4 on ECFCs functionality has not been studied before and it is also not known if ECFC’s BH4 level will affect cellular functionality in CAD patients. The present study therefore aimed to elucidate the effect of ECFC intracellular BH4, BH4/BH2 ratio on ECFC’s functionality in CAD patients.

## Materials and methods

### CAD patients and control subjects

Fifty-three patients of either sex between the age of 40–60 years with angiographically confirmed CAD having > 70% stenosis in left coronary artery were recruited from cardiac catheterization lab as cases and 42 age and gender matched healthy non diabetic, non-hypertensive subjects who either had normal angiogram or were negative on stress test (treadmill test; TMT) were recruited as controls. All subjects with past disease history of any form of cancer, cerebrovascular disease, renal disease, rheumatoid arthritis, pneumonia in past two weeks before blood collection, hormonal therapy and vitamin supplementation were excluded as these factors may influence the function and number of ECFCs. The experimental protocol was approved by institutional ethics committee and written consent was obtained from all the study subjects prior to recruitment for the study. The study was carried out in accordance with the guidelines of the Declaration of Helsinki. 30 ml of peripheral blood samples were collected in heparin tube from all the subjects at fasting state.

### Expansion of late EPC or endothelial colony forming cells (ECFCs) in in-vitro culture

ECFCs were cultured from peripheral blood of CAD patients and healthy controls. For the same peripheral blood mononuclear cells (PBMNCs), isolated from heparinized whole blood by ficoll density gradient centrifugation, were suspended in endothelial basal media supplemented with endothelial growth media (EGM) Single Quots (Lonza) and 10% fetal bovine serum (FBS). Mononuclear cells were then seeded onto rat tail collagen type-1 coated 6 well tissue culture plates (5 µg/cm^2^) at a seeding density of 1 × 10^7^/well and cultured at 37 °C and 5% CO_2_. Five days after seeding, non-adherent cells were aspirated out and adherent cells were further cultured and media was changed every other day until ECFC colonies appeared or up to a maximum of 60 days.

### ECFCs characterization

#### Flow cytometry phenotypic characterization

Phenotypic characterization of ECFCs were carried out at passage 2, in 05 representative samples from both CAD patients and healthy subjects, by flowcytometry. The expression of endothelial antigens CD31, KDR and CD146, progenitor antigens CD34, CD117, leucocytic antigen CD45 and monocytic antigen CD14 were assessed. Briefly, the adherent ECFCs were harvested by treating with Accutase cell detachment reagent (Cat. 561,527; Becton Dickinson) and then stained with 0.5 µL of FITC-conjugated mouse anti-human CD31 (Cat. 303,103; BioLegend), 0.5 µL of CD146 (Cat. 361,011; BioLegend), 1µL of PE-conjugated mouse anti-human KDR (Cat. FAB357P; R&D Systems) and 1 µL of CD117 (Cat. FAB332P; R&D Systems), 1 µL of APC-conjugated mouse anti-human CD34 (Cat. FAB7227A; R&D Systems), 0.5 µL of PE-Cy7-conjugated mouse anti-human CD14 (Cat. 557,742; BD Biosciences) and 2 µL of PECy5.5-conjugated mouse anti-human CD45 (Cat. 555,484; BD Biosciences). Cells were incubated with fluorophore conjugated antibodies at 4 °C for 30 min and stained cells were detected with Gallios flowcytometer (Beckman Coulter) and results were analysed with Kaluza software (Kaluza Analysis Software; version 2.1). ECFC characterization was carried out in representative samples of both CAD and control subjects. Cultured ECFCs were considered as negative for the leucocyte and monocyte marker CD45 and CD14, positive for the stem cell marker CD117, CD34 and positive for endothelial marker CD31, KDR, CD146 respectively.

#### Immunofluorescence characterization

Cultured ECFCs were assessed for KDR and CD34 expression in 05 representative samples from each CAD patients and healthy subjects. Briefly, cells at passage 2 with approximately 60–70% confluency were fixed with 4% paraformaldehyde (PFA) solution and blocked with 1% BSA. Cells were then incubated with mouse monoclonal anti-human KDR primary antibody (20 µg/ml) (Cat. MAB3571; R&D Systems) for 1 h followed by rhodamine conjugated goat antimouse secondary antibody (1:100 dil) (Cat. SR131, G-Biosciences) in dark for 1 h. Thereafter, cells were incubated with Alexa Fluor 488 conjugated mouse anti-human CD34 antibody (10 µg/ml) (Cat. FAB7227G; R&D Systems) for 1 h in dark. Counter staining was achieved by incubating cells with DAPI and images were obtained using upright Epi fluorescence microscope (Nikon Eclipse Ni with DSRi-2 colour camera).

In 05 representative ECFC samples from both CAD patients and healthy subjects, ability of cells to uptake acetylated low-density lipoprotein; Ac-LDL and Ulex-lectin binding was assessed. In brief, ECFCs at passage 2 were incubated with 1,1’-dioctadecyl-3,3,3’,3’-tetramethylindo-carbocyanine labelled Ac-LDL (Dil-Acy-LDL; 5 µg/ml) (Cat. L3484; Life Technologies), fixed with 4% PFA and then incubated with fluorescein isothiocyanate labelled Ulex europaeus agglutinin (FITC-UEA-1; 10 µg/ml) (Cat. FL-1061; Vector Laboratories). Cell nuclei were stained with 4’,6-diamidino-2-phenylindole (DAPI). Stained cells were observed on upright Epi fluorescence microscope (Nikon Eclipse Ni with DSRi-2 colour camera).

### Quantification of intracellular ECFC’s tetrahydrobiopterin (BH4) and dihydrobiopterin (BH2)

ECFC’s tetrahydrobiopterin, dihydrobiopterin and biopterin were quantified as described by *González *et al.^12^ with minor modifications. Briefly adherent cells at passage 3 for both CAD subjects and healthy controls were harvested and 1 × 10^6^ cells were used for biopterin estimation using high performance liquid chromatography (HPLC) (Agilent 1260 Infinity II) with fluorescence detector. The harvested cells were washed with ice cold phosphate buffer saline (PBS) in amber vial and then cell pellet was resuspended in 300 µl of ice-cold lysis buffer (composition: 50 Tris–HCl, 2 DTT, 1 EDTA mmol/L) and lysed by sonication using probe sonicator (4 cycles; 10 s each cycle at 100 W) on ice. The cell lysate was then centrifuged and supernatant was collected. Next, 25 µl of supernatant was used for protein estimation by Bicinchoninic Acid Assay (BCA) method. HClO4: H3PO4 was added to residual supernatant and vortexed vigorously for 5 s followed by 30 min incubation in dark at 4 °C for denaturation of protein in cell lysate. The cell lysate was then centrifuged at 13500xg for 5 min at 4 °C to pellet down the protein content and supernatant was collected for acid and alkali oxidation. For acid oxidation, 10 µl of acidic I_2_/KI (1% I_2_/2% KI Solution in 1 M HCl) was added to 90 µl of supernatant whereas 10 µl of 1 M NaOH and 10 µl of alkali I_2_/KI (1% I_2_/2% KI Solution in 1 M NaOH) were added to 80 µl of supernatant and then incubated for 1 h in dark. The alkali oxidized sample was then acidified with 20 µl of 1 M H_3_PO_4_ and both acid and alkali oxidized samples were then neutralized by adding 5 µl of 20 mg/ml ascorbic acid. After neutralizing, samples were filtered through 0.2 µm filter with 4 mm diameter under centrifugal force at 13500xg for 5 min at 23 °C. The filtered samples (50 µl) were then injected into C18 reverse phase analytical column. The total run time was 10 min with flow rate of 1 ml/minute of 5% methanol solvent system. Detection of biopterin was carried out at excitation and emission wavelengths of 350 and 450 nm respectively. Biopterins were quantified against standard curve of L-biopterin with 06 different concentrations (20–0.625 [picomoles/ml) was prepared by serial dilutions and % recovery of biopterin from BH4 and BH2 after chemical derivatization was assessed using standard BH4 and BH2 compounds. The chromatogram was obtained and peak area was analysed using Chemstation software, Agilent Technologies.

The quantification of BH4 in cell lysate was carried out from the difference between peak areas of acid and alkali oxidation, whereas BH2 was determined as the difference between the peak areas of alkali oxidation and samples without any oxidation process. The values of BH4, BH2 and biopterin levels were expressed in picomol per milligram of protein after normalization with the level of intracellular total protein.

### GTPCH mRNA expression by quantitative real-time PCR (qRT-PCR)

ECFCs at passage 4 in both CAD and control subjects were dissolved in 1.0 ml of TRIzol reagent (Ref. No.:15596018; Life Technologies, USA) and total RNA was isolated as per manufacturer instruction. The isolated total RNA was further dissolved in nuclease free water and quantified at 260 nm and 280 nm using NanoDrop (Thermo Scientific). Next for the cDNA synthesis, 500 ng of total RNA was reverse transcribed into cDNA using iScript cDNA Synthesis Kit (Cat. No: 1708891; BioRad). cDNA synthesis was carried out in Agilent Mastercycler (SureCycler 8800). For qRT-PCR, 3.0 µL cDNA, 15 µL master mix (Cat. No: 1725121; iTaq Universal SYBR Green Supermix; BioRad), 1.5 µL primers and 10.5 µL nuclease free water were pipetted into the appropriate strip tubes. Real-time PCR was performed on an Agilent AriaMx (G8830A AriaMx Real-Time PCR) with the following protocol; step 1: 1 cycle: for 30 s at 95 °C, step 2: 40 cycle: for 5 s at 95 °C and 15 s at 60 °C followed by continuous melt curve and melt peak analysis to ensure accuracy of amplified product. For each sample, runs were performed in triplicates. The primer sequences used to determine mRNA levels of GTPCH and housekeeping gene β Actin were: Forward 5' CGAGGAGGATAACGAGCTGA3', Reverse 5'CTGGTAGCCCTTGGTGAAGA3', Forward 5'AGAAAATCTGGCACCACACC3', Reverse 5'GGGGTGTTGAAGGTCTCAAA3'.

The relative quantification of gene expression was calculated by *∆∆*Ct method and the expression of target gene (GTPCH) was normalized with housekeeping gene; β Actin.

### ECFCs functional parameters

Functional assays including cell proliferation, adhesion, viability, in vitro wound healing and angiogenesis were carried out in duplicate in all samples from CAD patients and control subjects in which ECFC appeared, that is 27 CAD patients and 09 healthy control subjects. Angiogenesis assay was carried out in triplicates in a representative sample, that is 17 CAD patients and 09 healthy subjects.

#### Population doubling time

In vitro ECFCs proliferation was assessed by population doubling time (PDT) at passage 3. 1 × 10^5^ cells were seeded on collagen coated 35 mm culture dishes in duplicate and incubated at 37 °C with 5% CO_2_. After 72 h of incubation, cells were harvested and counted by neubauer chamber under phase contrast microscope. PDT of ECFCs was assessed using the formula: PDT = T ln_2_/ln (Xe/Xb), where T is time period (h), Xb is cell number at the beginning of incubation time and Xe is number of cells at the end of incubation time.

#### Expression of cell nuclear antigen Ki67

ECFCs proliferation was assessed through % expression of cell nuclear antigen Ki67 by flowcytometry. We investigated Ki67 expression in ECFCs of both CAD and control subjects at passage 3. Briefly, 1 × 10^5^ cells/well were seeded on a 6 well plate and incubated for 72 h at 37 °C, 5% CO_2_. After incubation, cells were harvested and permeabilized by treating with pre-chilled 70% ethanol and incubated for 1 h at − 20 °C. Thereafter, cells were resuspended into staining buffer and stained with PE conjugated Ki67 (Cat. 350,503, BioLegend) antibody and incubated for 1 h at room temperature. Cells were acquired in FACS LSR-II (Becton Dickinson). The unstained cell sample was used as negative control and Ki67-PE signal in logarithmic mode was recorded and data expressed as % expression of Ki67 in histogram.

#### Cell viability

Viability of ECFCs was monitored up to 7 days at 48 h interval post-seeding by performing the 3-(4,5-Dimethylthiazol-2-yl)-2,5-diphenyltetrazolium bromide (MTT) colorimetric assay. Briefly at passage 3, 1 × 10^4^ cells/well were seeded on collagen coated 96 well plate and incubated at 37 °C with 5% CO_2_. At 24 h and subsequent days with 48 h interval, cultures were incubated with MTT solution (12 mM) for 4 h at 37 °C. Then, the MTT solution was aspirated out and formazan crystals were solubilized with dimethyl sulfoxide. Absorbance of the dye was measured at a wavelength of 570 nm and recorded using a microplate reader (Synergy-2, Biotek).

#### Cell adhesion

ECFCs adhesion ability was assessed at passage 4, using method as described by *Oren M. Tepper *et al*.*^13^ with minor modifications. In brief, 1 × 10^5^ ECFCs/well were first seeded on collagen coated (5 µg/cm^2^ surface area) 24 well plate and incubated with EGM-2 for 1 h at 37 °C in 5% CO_2_. Adherent cells were then fixed with 4% PFA followed by staining with 0.1% crystal violet for 10 min, rinsing excess stain with water and addition of 200 µl of 10% acetic acid for elution of the stain. The absorbance of the eluted stain was measured at a wavelength of 600 nM with a microtiter plate reader (Synergy-2, Biotek).

#### Wound healing assay

In vitro wound healing assays were carried out using culture inserts as per the manufacturer’s instruction (IBIDI, Germany). Briefly, ECFCs at passage 4 were seeded at 2 × 10^4^ per well, in order to obtain confluent cell monolayer in 12 h, in two wells of the insert separated by a wall. Then culture inserts were removed creating a reproducible cell free gap of about 500 µm for each condition. The wound area filling was captured immediately, at 4 h and at 8 h by phase contrast microscope at 10 × magnification. The experiment was carried out in duplicate for all the samples and the microscopic images were analysed by T-Scratch software (TScratch version 1.0; Martin Schulz ETH Zurich) and data was expressed as percent of wound area at 0 h, 4 h and 8 h. The percentage of wound closure was calculated by using the formula as described by *Bronson I. Pinto*^14^; % of wound closure (X) = (i-f/i) × 100, where i = initial width of cell free gap at 0 h, f = final width of cell free gap.

#### Vasculogenesis assay

In vitro vasculogenesis or angiogenesis was assessed at passage 4 by plating 1 × 10^4^ cells on 10 µl of growth factor reduced matrigel matrix (Cat. 354,230; Corning) in µ-slides for angiogenesis (IBIDI, Germany). Cells were imaged using phase contrast microscope and 10 × objective after 12 h incubation at 37 °C and 5% CO_2_. This assay was performed in triplicate and number of tube junctions, number of branches of capillary tube and tube length were analysed using Image J (1.51 J8; National Institute of Health, USA) software with angiogenesis analyser plugin (http://rsb.info.nih.gov/ij/macros/toolsets/Angiogenesis%20Analyzer.txt) in 6 images per participant obtained from random selected microscopic field.

### Estimation of circulatory reactive oxygen and nitrogen species (ROS/RNS)

Estimation of total ROS/RNS in serum samples of both CAD and control subjects were carried out using OxiSelect in vitro ROS/RNS assay kit (Cell Biolab) as per the manufacturer instruction.

### In vitro treatment of SIN-1, a peroxynitrite generator, on ECFC viability assessed by MTT assay

At passage 2, ECFCs from healthy subjects (n = 3) were selected for the experiment. In brief, cells (1 × 10^4^/well) in 96 well plate were divided into control and treatment group. As described by *Yu Dong *et al*.*^15^ with minor modification, the cells in the five treatment groups were supplemented with 5 different concentrations of SIN-1 (00,221–1; Biotium) (200, 400, 600, 800, 1000 µM) with EGM-2 containing 1% FBS for 24 h. The effective concentration for subsequent experiments was determined by MTT. After, 24 h of treatment, MTT (12 mM) was added to each well and incubated for 4 h at 37 °C and the formazan crystals was dissolved in DMSO. The absorbance was measured at 570 nm and the cell viability was expressed as optical density with reference to the control.

### The in vitro effect of SIN-1 on ECFC’s intracellular biopterin levels

Estimation of intracellular ECFCs biopterin levels was performed after treatment with SIN-1. In brief, at passage 2, 1 × 10^5^ cells/ well were seeded in 6 well plate and incubated for 24 h at 37 °C, 5% CO_2_. Cells were then supplemented with starvation media containing EGM-2 with 1% FBS and incubated for overnight. In the treated group, SIN-1 was added in starvation media whereas for control, only EGM-2 with 1% FBS was added and incubated for 24 h. After treatment, estimation of biopterins were carried out by HPLC as described previously.

### ECFCs functionality after SIN-1 treatment in vitro

ECFCs functionalities in vitro were assessed in terms of cell proliferation, migration, in vitro wound healing and angiogenesis after treating the cells with SIN-1 for 24 h.

#### ECFCs proliferation as expression of cell nuclear antigen Ki67

The proliferation of ECFCs were assessed in terms of % expression of cell nuclear antigen Ki67 after SIN-1 treatment. Briefly, 1 × 10^5^ cells/well were seeded on 6 well plate. Cells underwent SIN-1 treatment for 24 h. After treatment, cells were stained with PE-conjugated Ki67 as described before and expression of proliferation marker Ki67 in SIN-1 treated and untreated samples was assessed by flowcytometry.

#### ECFCs migration

ECFCs migratory function was assessed in transwell migration assay as described by Tsai et al.^2^ with minor modifications. Briefly, EGM-2 medium supplemented with 10% FBS was added into the lower chamber, while 5 × 10^4^ cells in endothelial basal media (EBM-2) were seeded onto upper chamber of transwell permeable supports (8 µm pore size, polycarbonate membrane, SPL Life Sciences). Thereafter, cells were incubated for 4 h at 37 °C, 5% CO2. Suspension cells remaining in the upper chamber were removed and the migrated cells present in the lower side of the transwell insert were fixed with 4% paraformaldehyde. The migrated cells were next stained with DAPI and images from random selected fields and counted number of migrated cells using inverted fluorescence microscope (Nikon ECLIPSE Ts2RFL) and number of migrated cells were quantified and evaluated by Image J software (version 1.51 J8; National Institute of Health, USA).

#### The effect of SIN-1 treatment on in vitro wound healing by ECFCs

In vitro wound healing by ECFCs was assessed as described earlier after treating the cells with SIN-1 for 24 h. The experiment was carried out in duplicate and microscopic images at 0, 04 and 08 h were analysed using T-Scratch software (TScratch version 1.0; Martin Schulz ETH Zurich) and data was represented as percentage of wound closure at two different time points (0 to 04 h and 04 to 08 h).

#### The effect of SIN-1 treatment on in vitro vasculogenesis by ECFCs

In vitro vasculogenesis or angiogenesis was assessed by plating 5 × 10^3^ cells on growth factor reduced matrigel matrix (Cat. 354,230; Corning) in µ-slides for angiogenesis (IBIDI, Germany). Cells were imaged using phase contrast microscope after 06 h of incubation at 37 °C and 5% CO_2_. This assay was performed in triplicate and number of tube junctions, number of branches of capillary tube and tube length were analysed using Image J (version 1.51 J8; National Institute of Health, USA) software with angiogenesis analyser plugin (http://rsb.info.nih.gov/ij/macros/toolsets/Angiogenesis%20Analyzer.txt).

### Statistical analysis

Normality of the sample distribution of each variable was tested using Shapiro–Wilk’s test. Normally distributed continuous variables were expressed as mean with SEM and nonparametric data was represented as median with IQR values. For the categorical variables, results were presented in percentage and statistical significance level was computed by *Chi*^*2*^ test. Unpaired Student's test and Two-sample Wilcoxon rank-sum test were performed to compare difference in mean and medians respectively between the groups. Pearson’s and Spearman correlation were used for parametric and nonparametric parameters. Statistical significance level was considered as p-value less than or equal to 0.05. Stata 11.0 statistical software (Stata/SE 11.0 College Station TX, 77,845 USA) was used for data analysis and GraphPad Prism-8.0 for graphical representation.

### Ethics approval

The study was conducted after obtaining the approval from institute research ethics committee from our institute (All India Institute of Medical Sciences, New Delhi) (Ref. No.: IEC-47/09.12.2015, RP-3/2016).

### Consent to participate

Written informed consent was obtained from all the study participants. The informed consent was in accordance with the declaration of Helsinki.

### Consent for publication

All the authors has given consent for the publication of this article.

## Results

### Baseline characteristics

Demographic, clinical characteristics and baseline biochemical parameters of CAD and controls are summarized in Table 1. The mean age of CAD patients was 52.7 years and that of control subjects, 49.0 years. The mean BMI (body mass index) was significantly different between CAD and control groups. A significant proportion of CAD patients (87%) were on lipid lowering drugs and consequently cholesterol, triglyceride, HDL, LDL, VLDL were significantly lower in them compared to control subjects. Table 2 shows baseline characteristics of CAD case (n = 27) and controls (n = 9) in which successful ECFC cultures were established in vitro*.* Other than fasting glucose, which was significantly lower in controls*,* no significant differences were noted any of the baseline characteristics between CAD cases and controls.

**Table 1:** Demographic, anthropometric, medication history and biochemical variables in CAD cases and healthy controls.

Parameters	CAD Cases (n = 53)	Controls (n = 42)	*p* value
Age (years)	52.70 ± 5.4	49.00 ± 6.3	0.003
BMI (Kg/m^2^)	23.70 ± 4.1	26.07 ± 3.4	0.004
Gender (n %)	M (44; 83.0%) F (09; 16.9%)	M (34; 80.9%) F (8; 19.0%)	0.794
**Family history**			
CVD (n %)	11; 20.75%	06; 14.29%	0.414
Diabetes (n%)	19; 35.85%	08; 19.00%	0.071
Hypertension (n %)	30; 56.00%	5; 11.90%	< 0.0001
Dyslipidemia (n %)	02; 3.70%	07; 16.60%	0.033
**Lifestyle factors**			
Smoking (n %)	18; 33.96%	08; 19.0%	0.105
Alcohol (n %)	13; 24.53%	08; 19.0%	0.523
**Medication history**			
ACE Inhibitor (n %)	23 (43.4%)	Nil	
β-Blocker (n %)	45 (84.9%)	Nil	
Statin (n %)	46 (86.7%)	Nil	
Aspirin (n %)	47 (88.6%)	Nil	
Ca2 + Channel Blocker (n %)	08 (15.0%)	Nil	
Diuretics (n %)	10 (18.8%)	Nil	
Nitrate (n %)	38 (71.7%)	Nil	
**Baseline biochemical parameters**			
Fasting Blood Glucose (mg/dl)	111 (104—123)	105 (96—121)	0.084
Total Cholesterol (TC) (mg/dl)	126.0 ± 36.1	186.8 ± 49.3	< 0.0001
Triglyceride (mg/dl)	98.0 (65—133)	157.5 (121—213)	< 0.0001
HDL cholesterol (mg/dl)	36.0 ± 9.4	41.7 ± 9.0	0.003
LDL cholesterol (mg/dl)	76.1 ± 27.2	113.6 ± 32.6	< 0.0001
VLDL cholesterol (mg/dl)	12.0 (9.0 – 16.0)	24.6 (16.8 – 39.0)	< 0.0001
TC: HDL	3.56 ± 0.82	4.48 ± 0.84	< 0.0001
LDL: HDL	2.15 ± 0.68	2.72 ± 0.59	< 0.0001
Creatinine (mg/dl)	0.74 ± 0.18	0.71 ± 0.15	0.464

Data expressed as mean ± standard deviation for normally distributed data and as median and interquartile range (IQR) for non-normally distributed data. Categorical variables were represented in percentage. Unpaired t-test and Mann Whitney test U was performed for normally and non-normally distributed data and Chi2 test for categorical variables respectively. *p* values ≤ 0.05 was considered statistically significant.

**Table 2:** Demographic, anthropometric, medication history and biochemical variables in CAD cases and healthy controls: Based on successful ECFCs culture in vitro.

Parameters	CAD Cases (n = 27)	Controls (n = 9)	p value
Age (years)	51.62 ± 5.7	53.1 ± 7.0	0.532
BMI (Kg/m^2^)	24.5 ± 4.3	27.46 ± 4.4	0.088
Gender (n %)	M (23; 85.19%) F (04; 14.81%)	M (7; 77.78%) F (2; 22.2%)	0.475
**Family history**			
CVD (n %)	5; 18.52%	02; 22.22%	0.574
Diabetes (n%)	10; 37.04%	04; 44.44%	0.494
Hypertension (n %)	12; 44.44%	02; 22.22%	0.218
Dyslipidemia (n %)	02; 7.41%	Nil	
**Lifestyle factors**			
Smoking (n %)	08; 29.63%	02; 22.22%	0.514
Alcohol (n %)	04; 14.81%	02; 22.22%	0.475
**Medication history**			
ACE inhibitor (n %)	09 (33.3%)	Nil	
β-Blocker (n %)	22 (81.4%)	Nil	
Statin (n %)	23 (85.1%)	Nil	
Aspirin (n %)	23 (85.1%)	Nil	
Ca2 + channel blocker (n %)	03 (11.1%)	Nil	
Diuretics (n %)	03 (11.1%)	Nil	
Nitrate (n %)	20 (74%)	Nil	
**Baseline biochemical parameters**			
Fasting blood glucose (mg/dl)	114 (104–130)	89.8 (79.5—112)	0.021
Total cholesterol (TC) (mg/dl)	119.4 ± 40.2	144 ± 52.6	0.151
Triglyceride (mg/dl)	100 (58—148)	143 (103—179)	0.100
HDL cholesterol (mg/dl)	34 ± 9.9	36 ± 9.8	0.608
LDL cholesterol (mg/dl)	72.5 ± 31.1	85.7 ± 38	0.301
VLDL cholesterol (mg/dl)	11 (8.0 – 16.0)	21 (16.0 – 30.0)	0.012
TC: HDL	3.54 ± 0.83	3.9 ± 0.84	0.204
LDL: HDL	2.14 ± 0.74	2.36 ± 0.75	0.458
Creatinine (mg/dl)	0.71 ± 0.19	0.67 ± 0.17	0.655

Data expressed as mean ± standard deviation for normally distributed data and as median and interquartile range (IQR) for non-normally distributed data. Categorical variables were represented in percentage. Unpaired t-test and Mann Whitney test U was performed for normally and non-normally distributed data and Chi2 test for categorical variables respectively. *p* values ≤ 0.05 was considered statistically significant.

### In vitro ECFC culture and their expansion in CAD and control subjects

ECFCs appeared in culture with typical cobble stone morphology (Supplemental Fig. 1) similar to that of native endothelial cells. ECFC appeared in culture in 30 out of 53 patients with CAD (56%) and fourteen out of 42 control subjects (33%). Out of the 30 cases in which ECFCs appeared in culture, 03 did not proliferate adequately and no confluency was attained. Whereas similar limited proliferation was seen in 5 control samples.

### Characterization of ECFCs

#### Flowcytometric and immunofluorescence characterization:

ECFCs were characterized by flowcytometry and the average expression of endothelial markers CD31, CD146 and KDR were 88.8%, 88.2%, 52.2% in case of CAD whereas 87.6%, 89.1% and 57.1% respectively in case of control subjects. Whereas average expression of progenitor markers CD34 and CD117 were 36.08%, 28.2% and 46.2%, 31.2% respectively in case of CAD and control subjects. Panleucocyte (CD45) and monocytic marker (CD14) expression were negligible in both the groups (Supplemental Fig. 2).

ECFCs were positive for CD34 and KDR in immunofluorescent staining. ECFCs also showed Dil-Acy-LDL uptake and lectin binding (Supplemental Fig. 3).

### ECFC’s biopterins (BH4 and BH2)

**Figure 1:**
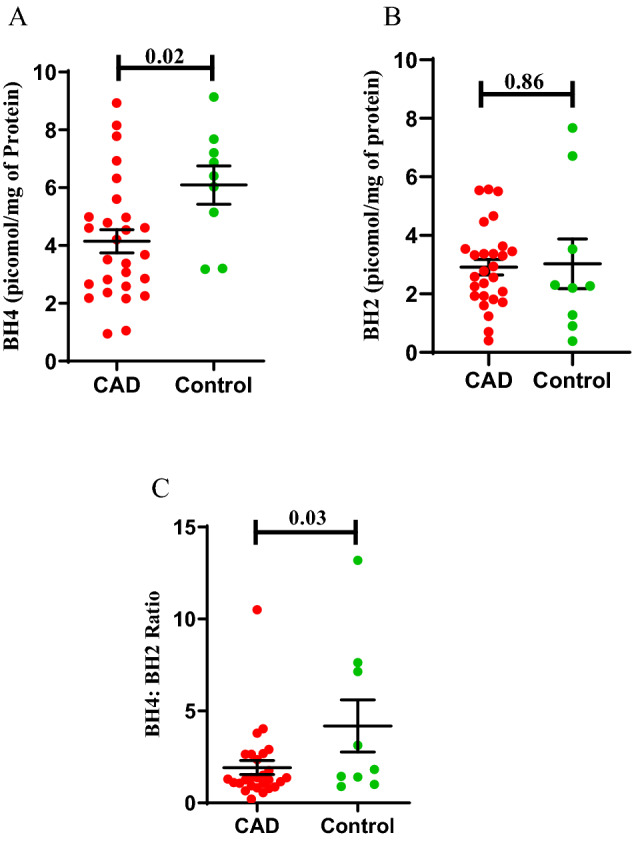
Intracellular ECFC’s Biopterin levels in CAD patients and controls. ECFC’s BH4 (Tetrahydrobiopterin), BH2 (Dihydrobiopterin), BH4/BH2 ratio in CAD (n = 27) and healthy control groups(n = 09). (**A**) ECFC’s BH4 in picomol/mg protein (**B**) ECFC’s BH2 in picomol/mg protein (**C**) BH4/BH2 ratio. Significance difference between the group was assessed by student t-test. Data is represented as mean ± SE. Error bar represent standard error of mean; dots represent average values of the parameters obtained from experimental duplicates; *p* ≤ 0.05 considered statistically significant.

Estimation of intracellular BH4 and BH2 levels were carried out in successfully expanded ECFCs from 27 CAD and 09 control subjects. The chromatogram of biopterins has been shown in supplemental fig. 4. The intracellular BH4 level was found to be significantly lower in ECFCs from CAD patients when compared to that of control subjects (4.14 ± 0.4; 6.09 ± 0.6; p = 0.02) (Fig. 1). The BH2 level however did not differ significantly between cases and controls (2.91 ± 0.2; 3.02 ± 0.8). BH4/BH2 ratio was also significantly lower in CAD patients compared to healthy controls (1.92 ± 0.3; 4.18 ± 1.4; p = 0.03).

### GTPCH mRNA expression

**Figure 2:**
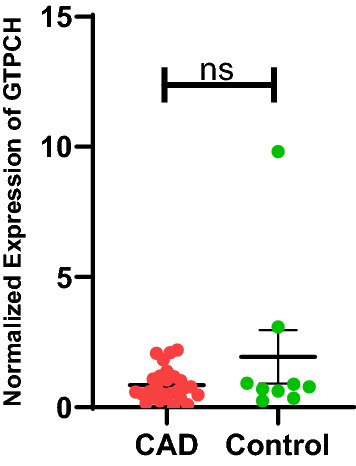
GTPCH mRNA expression in ECFCs isolated from CAD and healthy control. ECFC’s GTPCH mRNA expression level normalized with housekeeping gene β actin in CAD (n = 27) and control (n = 09) groups. Student t-test was used to assess the significance difference. Data is represented as mean ± SE. Error bar represent standard error of mean; dots represent average values of the parameters obtained from experimental triplicates. *p* ≤ 0.05 considered statistically significant.

Figure 2 shows the GTPCH-1 mRNA expression in CAD cases and controls. No significant difference in GTPCH-1 mRNA expression was observed.

### ECFCs functionalities

#### ECFC proliferation

**Figure 3:**
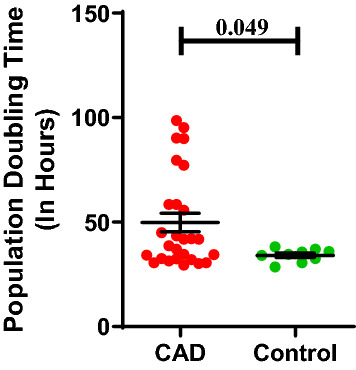
Population doubling time of ECFCs in CAD and healthy control subjects. Mean cell proliferation in terms of population doubling time (PDT) of ECFCs from CAD patients (n = 27) and control subjects (n = 09). In the histogram, Y axis represent PDT in hours and X axis represent the study groups (CAD and control group). Student t-test was used to assess the significance difference. Data is represented as mean ± SE. Error bar represent standard error of mean; dots represent average values of the parameters obtained from experimental duplicates; *p* ≤ 0.05 considered statistically significant.

**Figure 4:**
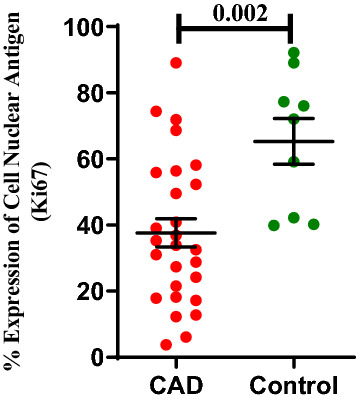
ECFCs proliferation depicted as % expression of cell nuclear antigen Ki67 in CAD and control group. Cell proliferation in terms of expression of Ki67 in ECFCs obtained from CAD and control group (n = 27; n = 09). In histogram Y axis represent rate (%) of expression of cell proliferation marker Ki67 and X axis represent the study groups (CAD and control group). Student t-test was used to assess the significance difference. Data is represented as mean ± SE. Error bar represent standard error of mean; dots represent average values of the parameters obtained from experimental duplicates; *p* ≤ 0.05 considered statistically significant.

Figure 3 shows the population doubling time (PDT) of ECFCs isolated from CAD subjects and healthy controls. PDT of ECFCs from CAD subjects was significantly prolonged compared to ECFCs from healthy control during 72 h of culture (49.8 ± 4.3; 34.1 ± 1.0; p = 0.049). The % expression of Ki67 as cell proliferation marker was significantly (p = 0.002) lower in ECFCs cultured from CAD patients as compared to control group (37.6 ± 4.2; 65.3 ± 6.9) (Fig. 4). Flowcytometry data acquisition for determination of ECFC’s Ki67 expression has been shown in supplemental figure 5.

#### Cell viability

**Figure 5:**
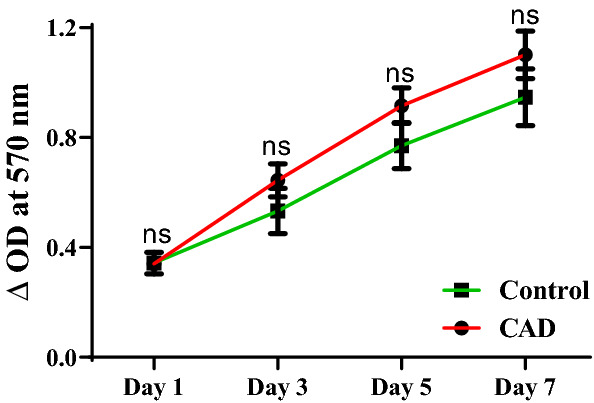
Cell viability assessed by MTT assay in CAD patients and controls. Mean cellular viability of ECFCs expressed in terms of change in optical density at different time points in CAD patients (n = 27) and control subjects (n = 09). Student ttest was used to assess the significance. Data is represented as mean ± SE. Error bar represent standard error of mean.

Cell viability was carried out by MTT assay and was found to increase from day 1 to day 7 in ECFCs from both CAD and control subjects with no significant difference between groups (Fig. 5).

#### Cell adhesion

**Figure 6:**
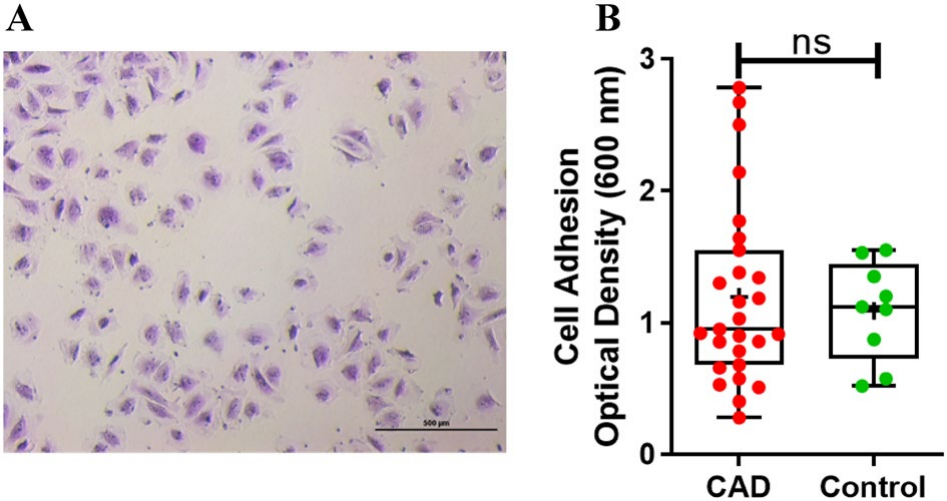
ECFCs adhesion ability in CAD cases and controls. (**A**) Brightfield microscopic image of ECFCs with crystal violet staining for cell adhesion. (10 × magnification; scale bar:500 µm). (**B**) Dot plot represent mean cell adhesion to collagen extracellular matrix in CAD and control groups (n = 27; n = 09). Significance was assessed by Mann–Whitney U test. Data is represented in Box-and-whisker plot with median (IQR), min/max.; dots represent average values of the parameters obtained from experimental triplicates; *p* ≤ 0.05 considered statistically significant. ns represent non-significant difference.

Cell adhesion ability of ECFCs did not differ between ECFCs from CAD and control subjects as shown in Fig. 6B. The microscopic image of ECFCs with crystal violet staining has shown in Fig. 6A.

#### In vitro wound healing

**Figure 7:**
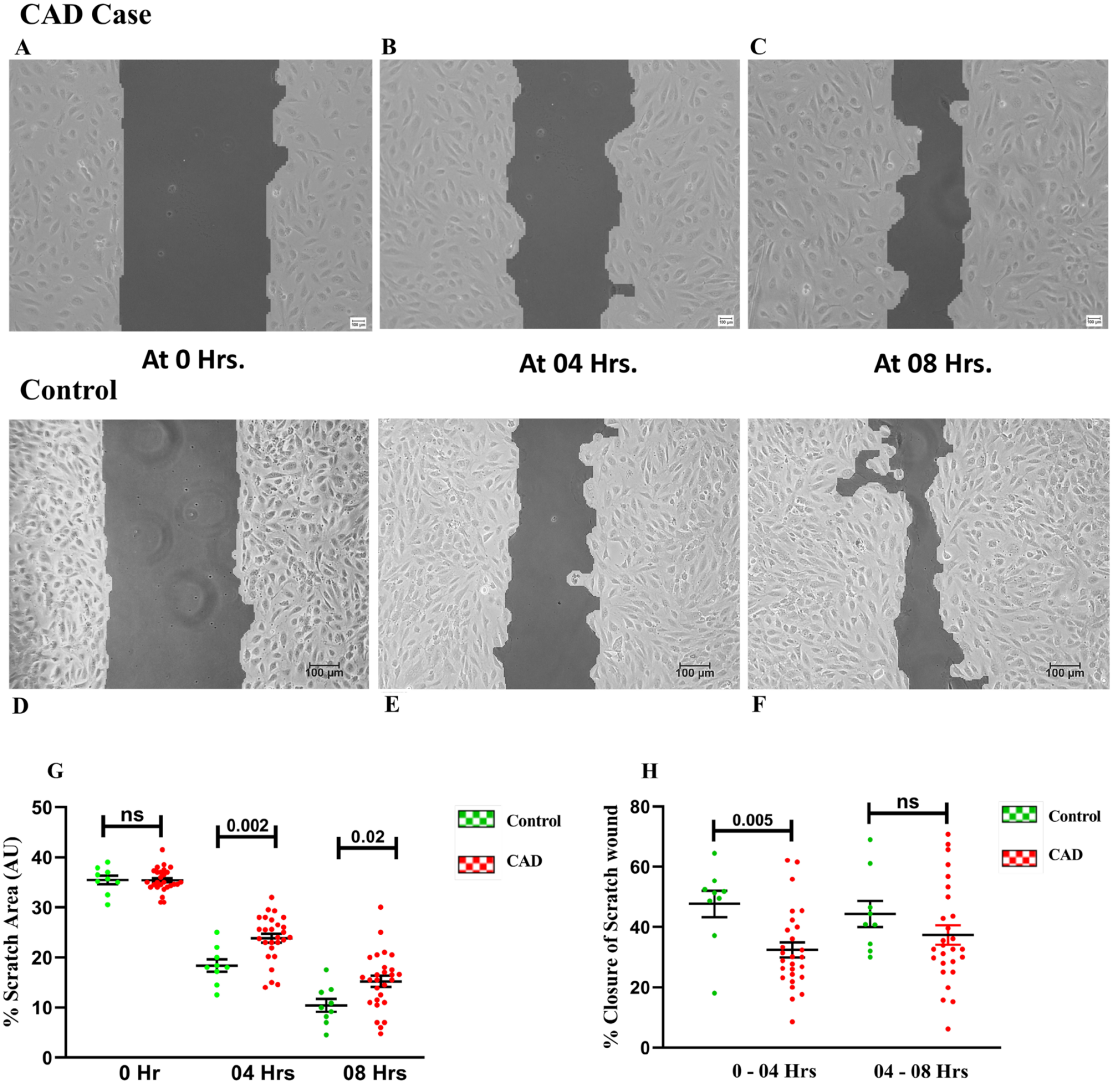
In vitro scratch wound assay by ECFCs at different time points in CAD and healthy controls. Bright field phase contrast microscopic images (10x) of in vitro scratch wound assay at different time points (0 h, 04 h and 08 h) is depicted in (**A**–**F)**. (**A**–**C)** shows 10 × image of wound closure that is 0 h, 4 h and 8 h in CAD patients (n = 27); (**D**–**F)** shows wound closure at three different time points that is 0 h, 04 h and 08 h in control subjects (**D**–**F**) (n = 09); Scale bar 100 µm; **G** shows histogram depicting % scratch wound area between the two-cell monolayer at three time points (0 h, at 04 h and at 08 h), (n = 27 for CAD and 9 for controls). (**H)** shows % in vitro scratch wound closure by ECFCs at two different time points from 0 to 04 h and 04 to 08 h in CAD (n = 27) and control (n = 9) groups (**H**). Student ttest was used to assess the significance difference. Data is represented as mean ± SE. Error bar represent standard error of mean; dots represent average values of the parameters obtained from experimental duplicates; *p* ≤ 0.05 considered statistically significant*.*

As shown in Fig. 7A–H, The scratch wound area at 04 h and 08 h was significantly different between CAD and control group with the CAD group showing larger wound area (Cell free area) as compared to control group (23.8 ± 0.88; 18.4 ± 1.2; p = 0.002, 15.2 ± 1.1; 10.4 ± 1.2; p = 0.02). Whereas, the wound regression at two different time points in terms of % closure of wound by ECFCs was significantly higher in the first 4 h in control group compared to CAD group (47.6 ± 4.3; 32.4 ± 2.5; p = 0.005). In vitro wound healing by ECFCs in terms of % wound regression at 4 h was found to be significantly (p = 0.04) associated with intracellular BH4 level (r = 0.4) (Supplemental Fig. 6A).

#### In vitro angiogenesis

**Figure 8:**
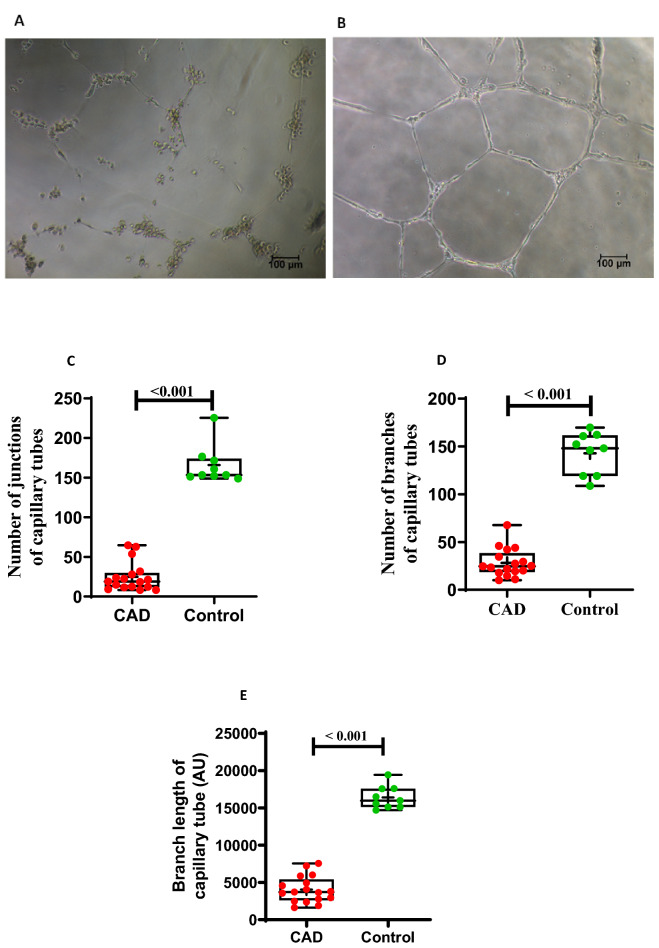
In vitro angiogenesis or capillary tube formation by ECFCs in CAD and healthy control group**. (A**) and (**B**) shows images of capillary tube formation by ECFCs on Matrigel matrix in a representative CAD and control sample respectively. Images are at 10 × magnification with 100 µm scale bar. (**C**) represents number of branches of capillary tubes in CAD (n = 17) and control group (n = 9). (**D**) depicts number of junctions of capillary tube in CAD (n = 17) and control (n = 9). (**E**) shows branch length of capillary tubes formed in cased (n = 17) and controls (n = 9). Box-and-whisker plot representing median (IQR), max/min and dots are average values; obtained from experimental duplicates. Significance was assessed between the group by Mann–Whitney U test. *p* ≤ 0.05 was considered statistically significant. AU: Arbitrary Unit*.*

Matrigel tubule assay was performed to investigate capacity of ECFCs to differentiate into tubule like structures. The number of branches of tube, length of branches and number of tube junctions were assessed in 17 and 09 representative ECFC samples from CAD and healthy control group respectively. All three parameters of in vitro capillary tube formation were significantly (p < 0.001) reduced in CAD patients compared to control group (Fig. 8A–E). In vitro angiogenesis by ECFCs by means of both number of capillary tube junctions and branch length of capillary tube were positively correlated with intracellular BH4 level (r = 0.54, p = 0.022; r = 0.51, p = 0.03) (Supplemental Fig. s6B & C).

### Circulatory total reactive oxygen and nitrogen species (ROS/RNS)

**Figure 9:**
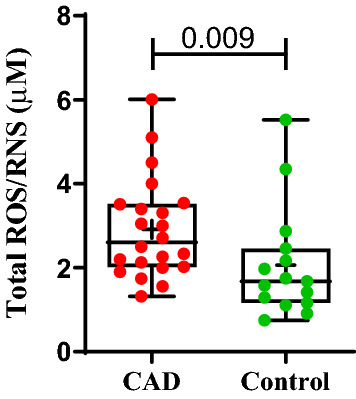
Total circulating ROS/RNS in CAD and control group. Box-and-whisker plot represents circulatory total ROS/RNS in CAD (n = 22) and control group (n = 15) with median (IQR), max/min and dots are average values of total ROS/RNS. Average values obtained from experimental triplicates. Significance between the groups was assessed by Mann–Whitney U test. Data is represented in Box-and-whisker plot with median (IQR), min/max.; dots represent average values of the parameters obtained from experimental triplicates; *p* ≤ 0.05 considered statistically significant.

Since the ROS/RNS is very unstable and highly susceptible to deterioration outside living system, the estimation was carried out only in few representative serum samples (CAD; n = 22, control; n = 15) stored at − 80 °C for not more than 60 days. The concentration of circulatory total ROS/RNS was measured in terms of µM H2O2 and was found to be significantly higher in CAD patients as compared to control subjects (p = 0.009; Fig. 9).

### Peroxynitrite donor SIN-1 induced ECFCs injury in vitro

MTT assay was performed to determine the cytotoxicity of different concentrations of SIN-1 on the ECFCs from healthy subjects. SIN-1(1000 µM) significantly (p < 0.0001) reduced cell viability when compared to that of untreated (Supplemental Fig. 7), so SIN-1 at this concentration was used to assess the effect of oxidative stress particularly nitrosative stress on intracellular biopterin levels and ECFCs functionalities including cell proliferation, migration, in vitro wound healing, angiogenesis.

### Intracellular biopterin levels and functionality of ECFCs after SIN-1 treatment

**Figure 10:**
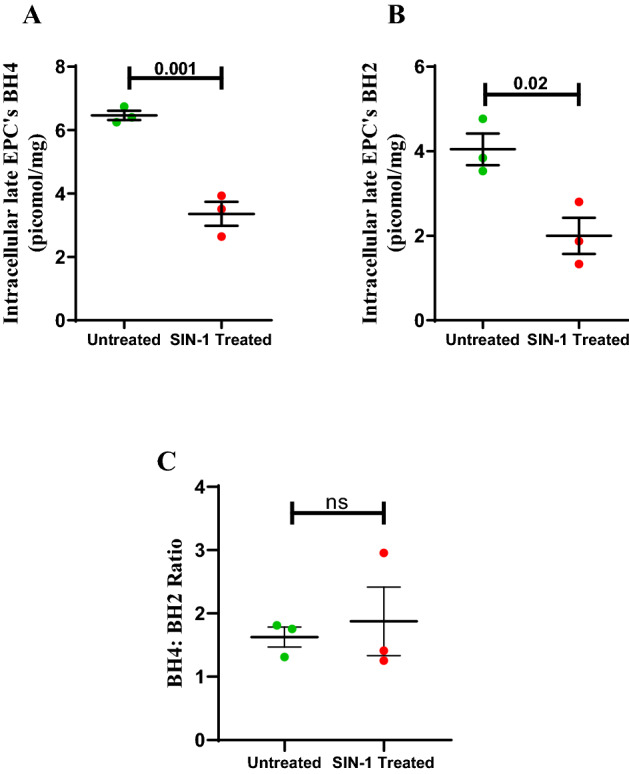
Effect of in vitro SIN-1 treatment on intracellular ECFC’s BH4, BH2 and BH4:BH2. Dot plot represents (**A**) intracellular ECFC’s BH4 (**B**) intracellular ECFC’s BH2 (**C**) BH4/BH2 ratio in untreated and SIN-1 treated groups with individual data points and error bars (n = 3 for each group). Significance was assessed by student ttest and data is represented as mean ± SE. Error bar represent standard error of mean; dots represent average values of the parameters obtained from experimental duplicates *p* < 0.05 was considered statistically significant.

The peroxynitrite donor SIN-1 treatment, significantly reduced intracellular BH4 as well as BH2 level when compared to that of untreated (p = 0.0016, p = 0.022; Fig. 10). However, there was no significant difference in BH4/BH2 ratio between the groups.

After treatment with SIN-1 for 24 h the ECFC’s when observed under bright field microscope showed a changed morphology with elongated shape whereas untreated cells showed the typical cobble stone morphology (Supplementary figure 8).

**Figure 11:**
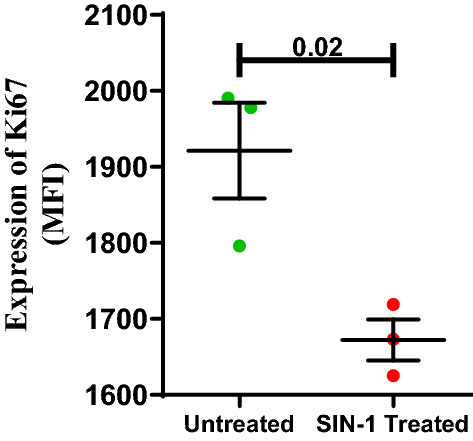
Effect of SIN-1 on ECFC’s Ki67 expression. Dot plot represents Ki67 expression of ECFCs in terms of median fluorescence intensity (MFI) in untreated and SIN-1 treated groups with individual data points and error bars (n = 3 for each group). Significance was assessed by student ttest and data is represented as mean ± SE. Error bar represent standard error of mean; dots represent average values of the parameter obtained from experimental duplicates p < 0.05 was considered statistically significant.

The effect of SIN-1 on proliferative potential of ECFCs, assessed in terms of expression of cell nuclear antigen Ki67, was found to be significantly p = 0.02) lower in SIN-1 treated ECFCs compare to untreated cells (Fig. 11).

**Figure 12:**
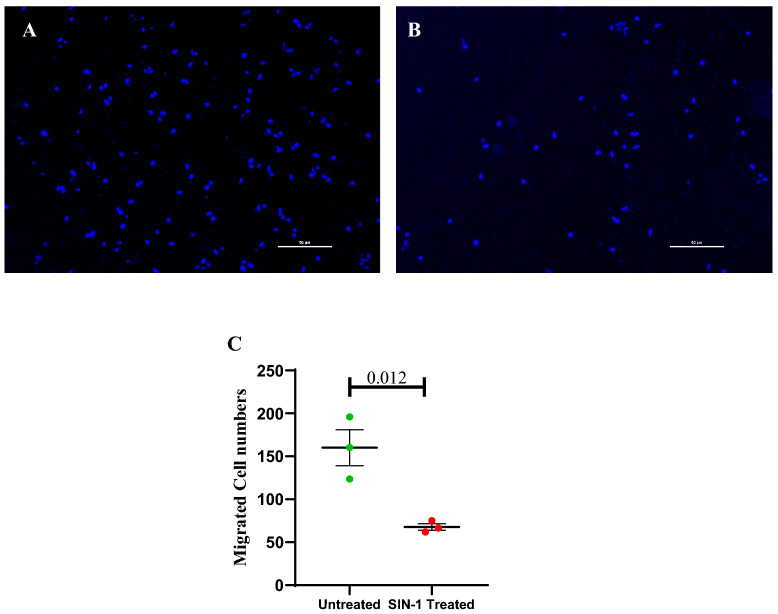
Effect of SIN-1 on ECFC’s migration**. (A**, **B**) represents transwell migrated cells with DAPI nuclear staining; (**A**) Untreated; (**B**) SIN-1 treated. Dot plot (**C**) represents number of migrated cells in lower chamber in untreated and SIN-1 treated groups with individual data points and error bars (n = 3 for each group). Significance was assessed by student ttest and data is represented as mean ± SE. Error bar represent standard error of mean; dots represent average values of the parameter obtained from experimental duplicates p < 0.05 was considered statistically significant.

After 24 h of SIN-1 treatment significantly lesser number of ECFCs migrated to the lower transwell chamber as compared to the untreated ECFCs (67.8 ± 3.8 vs 160 ± 20; p = 0.012) (Fig. 12).

**Figure 13:**
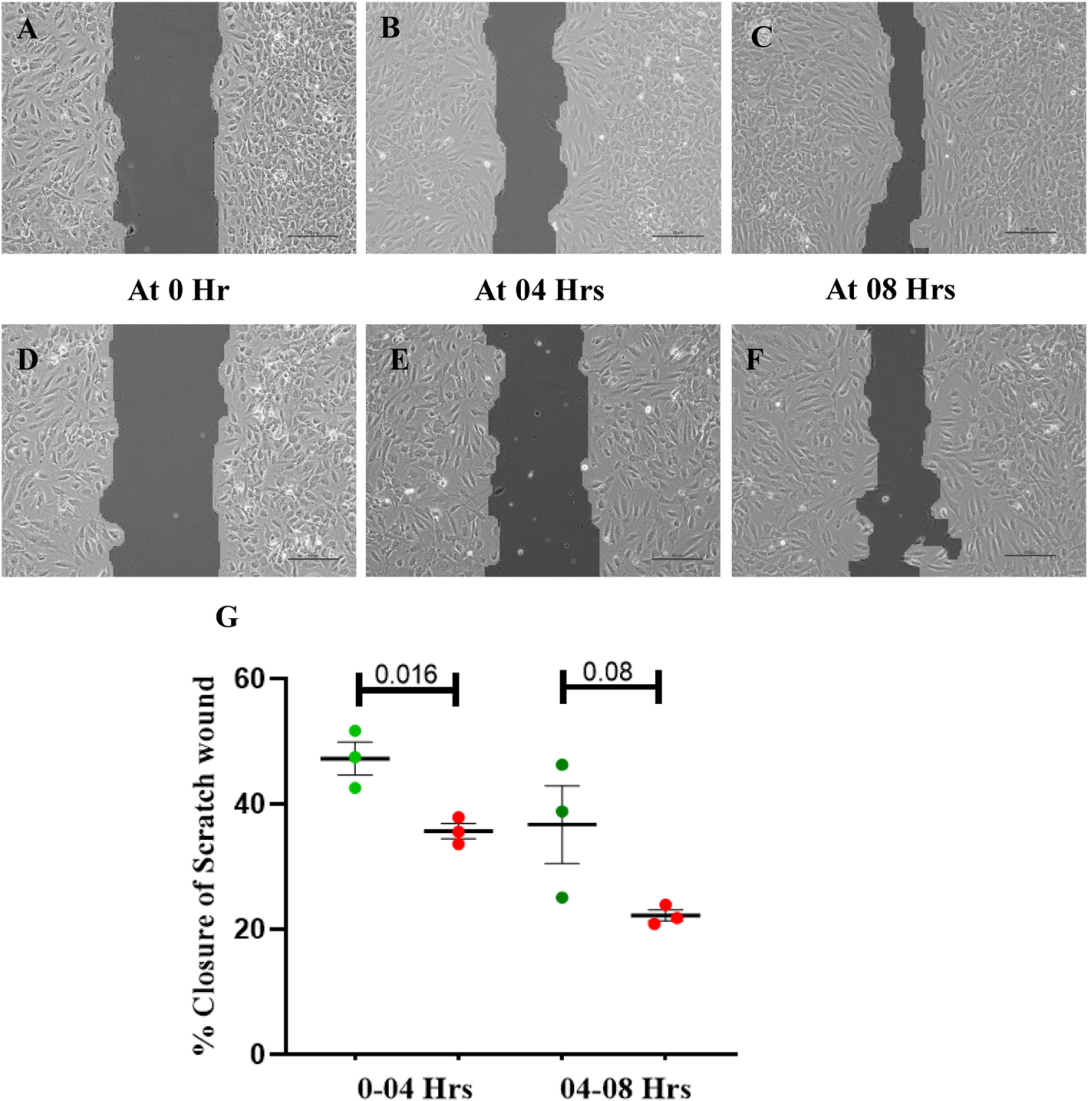
Effect of SIN-1 on scratch wound regression by ECFCs in vitro*.* Bright field phase contrast microscopic images (10x) of in vitro scratch wound assay at 03 different time points (0 h, 04 h and 08 h) is depicted in (**A**–**F**). Images (**A**–**C)** shows 10 × image of wound closure in untreated; (**D**-**F**) shows wound closure in SIN-1 treated group at three different time points 0 h, 04 h and 08 h; Scale bar 100 µm; (**H**) shows % in vitro scratch wound closure by ECFCs at two different time points from 0 to 04 h and 04 to 08 h in untreated and SIN-1 treated group (n = 3 for each group). Student t-test was used to assess the significance difference. Data is represented as mean ± SE. Error bar represent standard error of mean; dots represent average values of the parameters obtained from experimental duplicates; *p* ≤ 0.05 considered statistically significant.

In the in vitro scratch wound assay, the % wound regression in SIN-1 treated ECFCs was significantly lower as compared to that in untreated ECFCs (35.6 ± 1.2 vs 47.2 ± 2.6; p = 0.016) at 4 h. However, at 08 h the % wound regression in treated and untreated ECFCs were comparable (Fig. 13).

**Figure 14:**
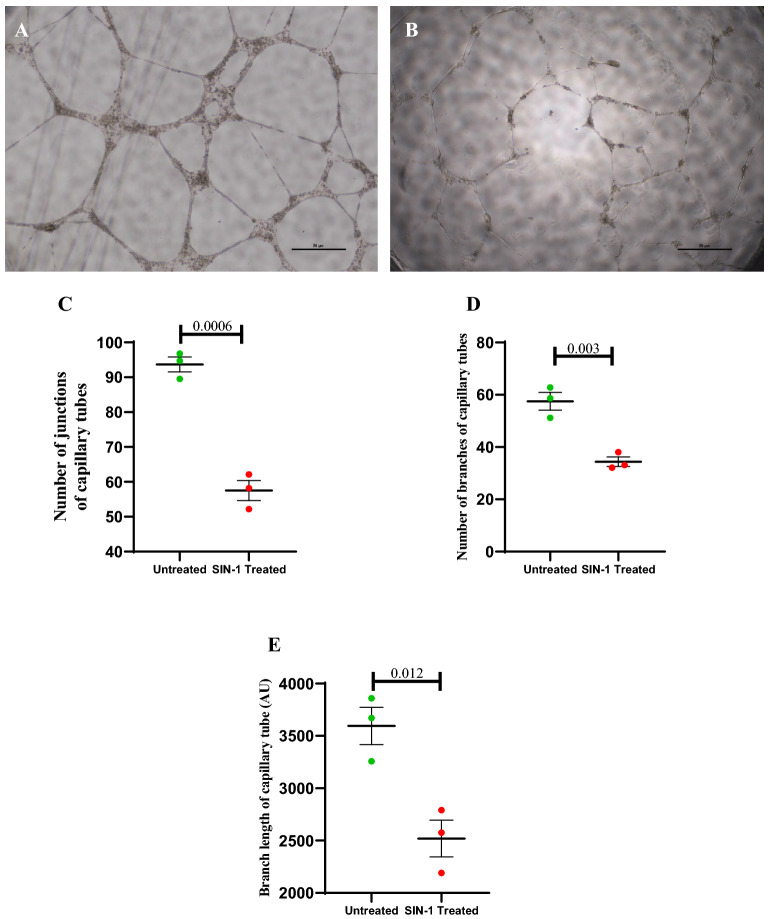
Effect of SIN-1 on in vitro angiogenesis by ECFCs. Image (**A**) and (**B**) shows images of capillary tube formation by ECFCs on Matrigel matrix in untreated and SIN-1 treated group respectively. Images are at 4 × magnification with 50 µm scale bar. (**C**–**E**) represents number of branches, number of junctions and branch length of capillary tubes formed in untreated and SIN-1 treated groups (n = 3 for each group). Student ttest was used to assess the significance difference. Data is represented as mean ± SE. Error bar represent standard error of mean; dots represent average values of the parameters obtained from experimental duplicates; *p* ≤ 0.05 considered statistically significant. AU: Arbitrary Unit.

In the in vitro 2D matrigel assay capillary tube formation was measured in terms of number of junction, number of branches and branch length of capillary tubes SIN-1 treated and untreated ECFCs. All three measurements of capillary tube formation were significantly reduced in case of SIN-1 treated ECFCs as compared to untreated. Data is shown in Fig. 14.

## Discussion

**Figure 15:**
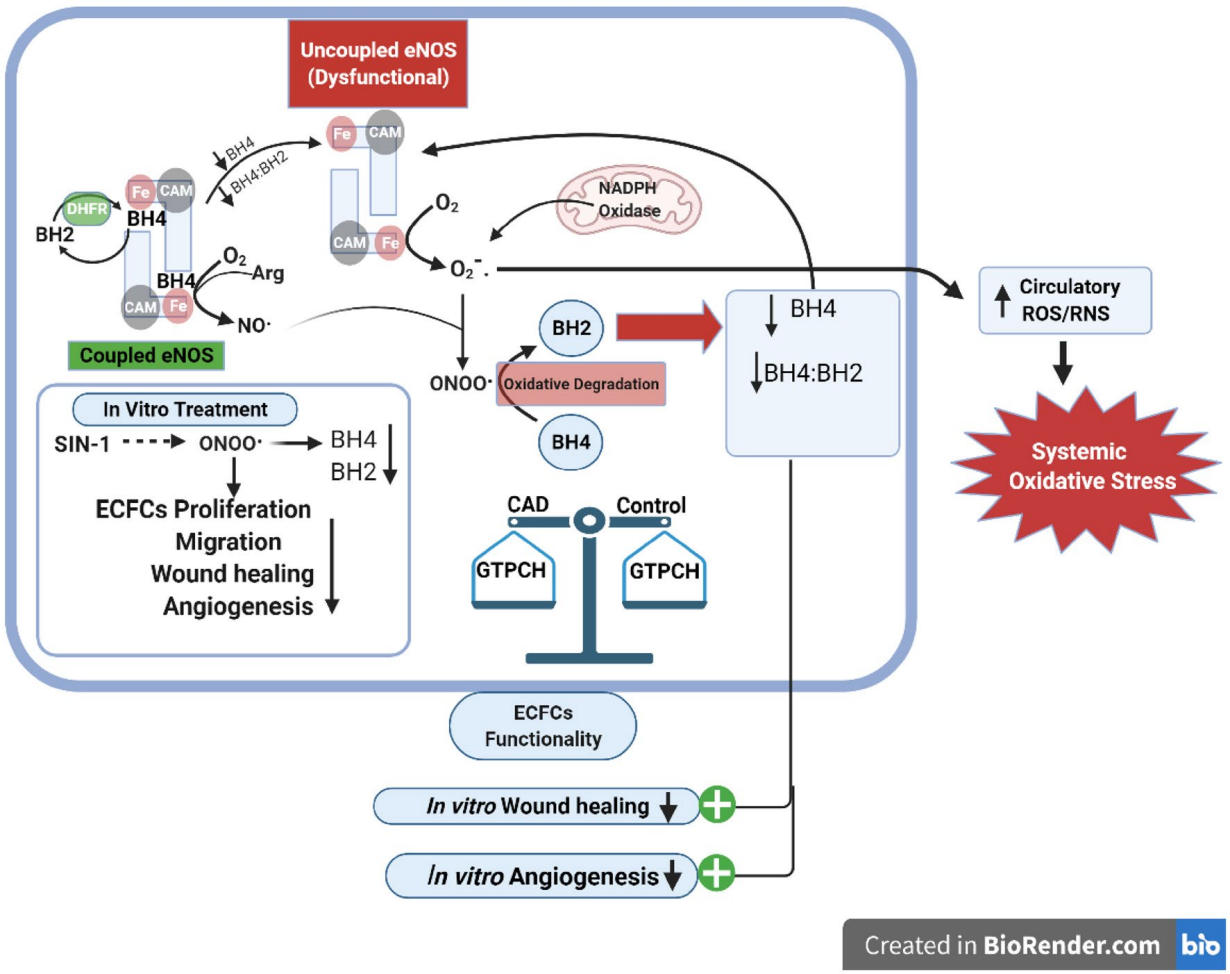
Graphical abstract of study findings. The illustration depicts the reduced intracellular ECFC’s BH4, ratio of reduced to oxidized biopterins (BH4:BH2) and their effect on cellular functionality in CAD patients. Generation of superoxide and peroxynitrite by NADPH oxidase and, eNOS uncoupling due to reduced BH4 levels may result in increased circulatory ROS/RNS in CAD patients. In vitro negative effect of peroxynitrite generator SIN-1 on ECFC’s biopterins (BH4, BH2) and cell functionalities as assessed by cell proliferation, migration, wound healing and angiogenesis. In CAD, ECFC’s BH4 biosynthesis might not be affected as rate limiting enzyme GTPCH mRNA expression was comparable with control..

In the current study we looked at the correlation of ECFC tetrahydrobiopterin levels with its functionality in CAD patients and healthy controls. We observed that ECFC from CAD patients were less proliferative as evidenced by lower Ki67 expression and prolonged population doubling time, and showed impairment in migration as seen by delayed wound healing capacity in vitro and significant decline in angiogenesis potential as compared to ECFCs from healthy controls. A significant association between ECFC BH4 levels and its wound healing capacity and angiogenesis potential was observed. An elevated circulatory ROS/RNS levels was evidenced in CAD patients pointing to peripheral oxidative stress. Oxidative stress induced in ECFC in vitro with SIN-1 was associated with reduction in BH4 levels and decreased proliferation, migration and angiogenesis. A concise graphical abstract of the present study findings has been illustrated in Fig. 15.

An impairment in early EPC functionality in terms of their adhesion and migration capacity has been reported in diabetic and hypertensive patients^13,16–18^. Studies on functional capacity of late EPCs or ECFC, which are the true functional EPCs is limited. *Kevin Tan *et al*.* found that ECFCs from patients with proliferative diabetic retinopathy (PDR) were impaired in their ability to migrate towards SDF-1 as well as human sera. Also, ECFCs from patients with PDR showed impaired ability to incorporate into and form vascular tubes with human retinal endothelial cells^19^. In contrary, *John D. Stroncek *et al*.* reported no significant difference in population doubling time of late outgrowth EPCs in CAD and healthy controls, PDT was however assessed after thawing of cryopreserved cells. The authors also reported no significant difference in matrigel tube formation by late out growth EPCs between CAD and healthy control groups. The study was however limited by very small sample size^20^. In another study on ischemic cardiomyopathy patients no difference in angiogenic potential of blood outgrowth endothelial cells was reported between patients and healthy subjects^21^ however the patient group comprised of stable ischemic cadiomyopathy as compared to patients in our study who had coronary artery disease. To the best of our knowledge, no study has comprehensively looked at all the four functional assays that is in vitro adhesion, migration, proliferation and angiogenesis assays in ECFCs in CAD patients. Assessment of all functional aspects of ECFC, that is proliferation or the ability of EPC to expand after vascular damage or tissue ischemia, adhesion of ECFCs to endothelium for re-endothelization and angiogenesis, migration of ECFC through the extracellular matrix for growth of new vessels and finally the ability of ECFC to form vascular structure, is important to understand the global function of ECFC’s in a disease state^22^.

Our finding of a reduced ECFC BH4 in CAD patients as compared to healthy controls point to either an oxidative reduction of BH4 to BH2 levels due to reactive oxygen and nitrogen species in patients or impaired biosynthesis of BH4. We did not find any significant difference in the GTPCH-1 expression between CAD patients and controls. We did find an increase in circulating ROS/RNS in CAD patients compared to controls. The effect of oxidative stress particularly nitrosative stress was confirmed by treating ECFCs with SIN-1, a peroxynitrite generator, which resulted in a reduced BH4 further confirming the role of nitrosative stress on determining intracellular biopterin levels. The reduced BH4 level and imbalance in the ratio of reduced to oxidized biopterin in the present study may lead to uncoupling of eNOS enzyme in ECFCs of CAD patients. In vivo, in vitro and clinical study have shown the transformation of eNOS from protective enzyme to a contributor of oxidative stress^23^. In line with our finding of increased peripheral oxidative stress , *da Cunha *et al*.* have postulated that, eNOS uncoupling is one of the source of systemic level of oxidative stress which was explained by high plasma lipoperoxidation levels in monosodium glutamate (MSG) induced obese rats compared to control and treatment with eNOS inhibitor *N*^G^-nitro-L-arginine methyl ester (L-NAME) was able to reduce the systemic lipoperoxidation levels in both obese rats as well as controls^24^. Similar to our observation of a reduced BH4 levels, its possible effect on eNOS catalytic activity and consequent effect on ECFC proliferative capacity, migration capacity and angiogenesis potential with SIN-1 treatment, *Yu Dong *et al*.* reported a decline in phosphorylation of eNOS and in vitro tube formation by EPCs in SIN-1 treated group^15^. We also observed a reduced BH2 levels with SIN-1 treatment which possibly reflects impaired BH4 biosynthesis. Both oxidative loss of BH4 and reduced biosynthesis of BH4 may be acting to determine intracellular biopterin levels.

The strength of our study is that we have studied all functional aspects of ECFCs which is important to understand global function. Secondly, we have done our study in ECFCs which are true cells of endothelial lineage unlike early EPCs on which most studies have been conducted. Thirdly we have also confirmed the role of nitrosative stress on biopterin levels in ECFCs in vitro. We also note some limitations. We did not measure the catalytic active state of eNOS to conclusively state that the reduced BH4 levels are causing uncoupling of eNOS. Further we have also not demonstrated the direct effect of augmenting BH4 levels on ECFCs functionality.

In conclusion, present study suggests the role of intracellular ECFC’s tetrahydrobiopterin as well as reduced to oxidized biopterin ratio in maintaining the ECFC functionality in CAD patients. Although we have looked at ECFC’s GTPCH-1 expression, the rate limiting enzyme of BH4 biosynthetic pathway in CAD patients, it is also imperative to look at expression of DHFR which is the rate limiting enzyme of the salvage pathway for regeneration of BH4. This will help to understand whether the reduced level of BH4 is contributed by oxidative degradation, by reduced biosynthesis or impaired regeneration through salvage pathway. Future studies should also be done to demonstrate the involvement of eNOS activity with lower BH4 levels in ECFC functionality.

Since BH4 regulatory system itself offer multiple pharmacological targets for therapeutic prevention of endothelial dysfunction and the progression of cardiovascular diseases a combinatorial approach of antioxidant therapy and BH4 supplementation might be useful in treatment of cardiovascular diseases^25^. Hattori et al. showed positive effect of in-vitro treatment of statin on GTPCH-1 expression in mature endothelial cell which resulted in increased levels of BH4 and NO^26^. In another study, Meyer et al. reported improved functionality of cord blood ECFCs with in-vitro treatment of pravastatin^27^. Our CAD patients were on statins but inspite of this the BH4 levels were lower in patients. A properly conducted randomized control trial can only demonstrate the effect if any of statins on ECFC BH4 levels in-vivo. The present study shows the in vitro effects of BH4 depleted ECFCs however, the angiogenic potential of BH4 depleted ECFCs from CAD should be tested further in preclinical model to reproduce the in vitro observations.

## Supplementary Information

Supplementary Figures.
