# Supplementary material for: Role of endothelial colony forming cells (ECFCs) Tetrahydrobiopterin (BH4) in determining ECFCs functionality in coronary artery disease (CAD) patients
Source: Sci Rep. 2022 Feb 23;12:3076. doi: 10.1038/s41598-022-06758-8 (PMC8866483; doi:10.1038/s41598-022-06758-8)
Supplement: Supplementary file 1 — Supplementary Figures. [file 41598_2022_6758_MOESM1_ESM.docx]

**Supplementary Data**

**Supplementary figure S1:** **Phase contrast microscopy images of ECFCs**

*Image (A) and (B) depicts ECFC colony isolated from healthy control on the day of appearance at 10x and 20x magnification respectively; (C) & (D) depicts ECFC culture at approximately 80% confluent viewed with 10x and 20x magnification respectively; (E) Confluent culture with typical cobble stone morphology at 10x magnification; (F) Cells with limited or no proliferative potential with elongated morphology, black arrows show limited proliferative EPCs with elongated morphology at 10x magnification. Scale bar: Images A to E 100µm, image E; 50µm.*

**Supplementary figure S2:** **Expression of cell surface markers in ECFCs in CAD (A) and control subjects (B)**

*Histogram of flowcytometric immunophenotypic characterization based on cell surface markers including endothelial markers (CD146, CD31 and KDR), progenitor markers (CD34 and CD117) and leukocytic and monocytic markers (CD45 and CD14) in a representative CAD subject. Left panel shows unstained sample and right panel shows expression of specific cell surface markers. The scale represents positive expression of markers with percent expression. In histogram, X axis represent fluorescence signal in log scale and Y axis represent count of events.*

*Histogram of flowcytometric immunophenotypic characterization based on cell surface markers including endothelial markers (CD146, CD31 and KDR), progenitor markers (CD34 and CD117) and leukocytic and monocytic markers (CD45 and CD14) in a representative control subject. Left panel shows negative control as unstained sample and right panel shows expression of specific cell surface markers. The scale represents positive expression of markers with percent expression. In histogram, X axis represent fluorescence signal in log scale and Y axis represent count of events.*

**Supplementary figure S3:** **Immunofluorescent staining of ECFC**s

1. **Immunofluorescent staining of cell surface markers CD34 and KDR**

*Immunofluorescence microscopy images of ECFCs isolated from healthy subjects for progenitor marker and endothelial marker. (A) DAPI for nuclear stain; (B) Endothelial marker KDR (VEGFR-2); (C) Progenitor marker CD34; (D) Merged image. All the images are with 20x magnification. Scale bar: 100µm.*

1. **Uptake of Acy-LDL and Lectin binding by late EPCs**

*Fluorescence microscopy images for Dil-Acy LDL uptake and FITC-UEA lectin binding by ECFCs. (A) DAPI for nuclear staining; (B) Dil-Acy LDL uptake; (C) UEA lectin binding; (D) Merged image. All the images are with 20x magnification. Scale bar: 100µm.*

**Supplementary figure S4:** **Chromatogram of standard biopterin and derivatized biopterins from BH4 and BH2 in cell lysate**

*Representative chromatograms of biopterins derived from cell lysates of healthy subjects. The Y axis represent fluorescence unit; X axis represent retention time of the biopterin molecule. (A) Standard biopterin at a concentration of 10 picomol/ml; (B) Biopterin in cell lysate without chemical derivatization; (C) Biopterins, including BH2 and biopterin after alkali oxidation; (D) total Biopterins, including biopterins + BH2 + BH4 after acid oxidation. The arrows represent chromatogram peak of biopterin.*

**Supplementary figure S5:** **Flowcytometric determination of nuclear antigen Ki67 in ECFCs**

*Flowcytometric determination of cell proliferation in terms of percentage of expression of cell nuclear antigen Ki67. (A) In scatter plot side scatter versus forward scatter, the gate P1 shows the cells of interest and exclusion of cell debris in unstained sample. (B & C) Histogram plot of unstained and Ki67 stained sample, gate P4 represent count of event for negative population and events in gate P5 represent positive population for Ki67 in a representative CAD sample (D & E) Histogram plot of unstained and Ki67 stained sample, gate P4 represent count of event for negative population and events in gate P5 represent positive population for Ki67 in a representative control sample.*

**Supplementary figure S6: Correlation between ECFC’s BH4 and *in vitro* wound healing and *in vitro* angiogenesis**

*Spearman correlation between intracellular ECFC’s BH4 and ECFCs functionality in terms of in vitro wound healing and in vitro angiogenesis. (A) Intracellular BH4 significantly correlated with % wound regression (n = 27). (B) BH4 significantly correlated with number of capillary tube junctions and (C)branch length of capillary tube (n = 17). p ≤ 0.05 was considered statistically significant. AU: Arbitrary Unit.*

**Supplementary figure S7:** **Effect of SIN-1 on ECFCs viability**

*Bar graph represents % of cell viability at different concentration of SIN-1 treatment including untreated (n=3). Cell viability (%) in each concentration of SIN-1 was calculated against untreated. Significance difference between the group was assessed by student ttest. Data is represented in mean ± SD and p≤0.05 was considered statistically significant; (****) represents p≤0.000.*

**Supplementary figure S8: Effect of SIN-1 on ECFCs morphology**

*Bright field microscopic Images (A) and (B) depicts ECFCs morphology at 10x and 20x magnification whereas images (C) and (D) represents ECFCs after SIN-1 treatment with elongated cell morphology at 10x and 20x magnification. Scale bar: Images A to E 100µm, image E; 50µm.*
